# Supplementary material for: Are carbon emissions trading and green financial instruments synergistic? -Comprehensive quantitative research based on content analysis
Source: PLoS One. 2024 Mar 7;19(3):e0298601. doi: 10.1371/journal.pone.0298601 (PMC10919601; doi:10.1371/journal.pone.0298601)
Supplement: S1 Data — (ZIP) [file pone.0298601.s001.zip › Data/地级市Z_F工作报告.zip!/║╙─╧/Σ≡║╙╩╨/└┤╫╘╣1⁄2╓┌║┼í╛┬φ┐╦╩2╛▌═°í┐_2018.docx]

各位代表：现在，我代表市人民政府，向大会报告工作，请予审议，并请各位政协委员和其他列席人士提出意见。

一、过去一年工作回顾

2017年，在省委、省政府和市委正确领导下，在市人大、市政协监督支持下，全市上下以迎接党的十九大胜利召开和学习贯彻党的十九大精神为动力，以习近平新时代中国特色社会主义思想为指导，全面贯彻新发展理念，坚持稳中求进工作总基调，突出供给侧结构性改革主线，统筹抓好稳增长、促改革、调结构、惠民生、防风险各项工作，经济运行总体平稳、稳中有进、进中向好，主要指标增速高于全省平均水平，较好地完成了七届人大一次会议确定的目标任务。

（一）发展质量持续提升。初步核算，全市完成生产总值1165亿元，增长8.2%；规模以上工业和服务业增加值分别增长8.3%和10.3%；固定资产投资增长11.8%；社会消费品零售总额增长12%；一般公共预算收入82.6亿元，增长12.3%；城乡居民人均可支配收入20750元，增长9.9%。其中，二三产业增加值占生产总值比重达到90.2%，高新技术产业增加值占规模以上工业增加值比重达到35.7%，规模以上工业利税占主营业务收入比重达到10.5%，税收占一般公共预算收入比重达到76.4%。

（二）经济转型持续加快。着力推动产业升级。出台产业转型发展攻坚总体方案和六个专项推进方案，启动工业扩量提质转型升级行动，推进“十百千”亿级企业及产业集群培育工程和“小升规”工程。新入库规模以上工业企业59家，全市149家企业实施技改项目182个，2家企业成为国家两化融合管理体系贯标试点。深入实施加快现代服务业发展行动，新入库规模以上服务业企业103家，服务业增加值占比达到28.9%，同比提高1.8个百分点。加快推进农业现代化，在受灾之年、玉米种植面积调减14.8万亩的情况下，粮食总产仍达到177.8万吨；新建高标准粮田12万亩，新发展、改善有效灌溉面积27万亩；农业综合机械化水平达到86.9%；新筹建农业产业联盟12个，农业产业化集群达到21个，市级以上农业产业化龙头企业达到121家。着力扩大有效需求。扎实开展重点项目攻坚活动，36个省重点项目完成投资244亿元，165个市重点项目完成投资516亿元，均超额完成年度计划。争取上级政策性资金90亿元、争取转贷地方政府债券21亿元。新增贷款99.7亿元，按可比口径增长15.5%。7个PPP项目签约落地、投资总额75.9亿元，其中2个项目被评为财政部示范项目。消费对经济的拉动作用进一步增强，假日经济持续升温，住房、汽车、信息等重点领域稳定增长，电子商务、邮政快递等新业态新模式快速发展，商品房销售面积增长49.9%，电商交易额突破百亿元，快递业务收入增长93.4%，我市成为国家快递示范城市创建城市。着力发展实体经济。完善企业服务机制，一企一策研究扶持措施，着力降低实体经济企业成本，为小微企业减免增值税1.5亿元，惠及纳税人14.4万户次。夯实实体经济发展平台，全市产业集聚区实现主营业务收入2987亿元，增长13.6%；服务业“两区”累计完成投资71.2亿元、增长22%，实现税收6.6亿元、增长51.9%。在全省产业转型攻坚暨重点项目观摩评价活动中，我市荣获综合排名小组第二名，经开区获得“十强产业集聚区”，源汇区特色商业区被评为全省“十先”，舞阳、郾城特色商业区晋级为全省一星级服务业“两区”；漯河临港产业园、中国（漯河）电子商务产业园获批全省首批服务业专业园区；临颍县荣获全省首批产业集群商标品牌培育基地。我市被评为全省唯一的2017年“全国质量魅力城市”。

（三）发展动能持续增强。坚持全面深化改革。扎实推进“放管服”改革，取消、下放行政审批事项102项，清理规范行政审批和中介服务82项、行政事业性收费41项、经营服务性收费14项；全面实行“三十五证合一”“一照一码”，创新个体工商户线上登记新模式，实现企业登记“零见面”“零介质”“零收费”全程电子化，新登记各类市场主体2.3万户。率先启动跨部门“双随机、一公开”联合抽查，经验在全省推广。统筹抓好各项重点改革，投融资体制改革稳步推进，农信社改革取得重大突破，不动产登记改革不断深入，城市管理执法体制改革圆满完成，国有非工企业改革稳妥展开。财税体制改革顺利实施，“四大行业”营改增入库税收10.4亿元，减税达1.08亿元。公共资源交易平台实现省市县三级联通，政务服务平台建成运营，753项权力事项全部实现网上办理。坚定不移扩大开放。河南自贸区漯河协同发展区率先获批，成功举办第十五届中国（漯河）食品博览会。全年签约投资超亿元项目104个，投资总额686.3亿元；实际利用外资9.01亿美元，实际利用省外资金240.7亿元；进出口总值52.8亿元，增长19%。着力实施创新驱动。全市科技支出4.13亿元，同比增长2.12倍。安排“科技券”专项资金，激发企业创新活力，专利授权量1270件、增长44.5%。新增国家级高新技术企业10家、省级工程技术研究中心4家、省级众创空间和科技企业孵化器各1家、省级星创天地2个，全市首家省级重点实验室“河南省天然色素制备重点实验室”获批建立。

（四）城乡面貌持续改观。深入开展全国文明城市、国家卫生城市创建，全面启动百城建设提质工程，我市被确定为全国城市“双修”试点市和城市设计试点城市，成功摘取“国家卫生城市”桂冠。加强规划统筹。进一步完善规划体系，编制完成生态水系、生态绿城规划纲要，基本实现城市规划全覆盖。积极推动规划工作创新，修订出台规划委员会工作规则和控规动态维护、城市规划技术管理规定，对规划领域部分事权和规划审批流程进行优化，着力破解城市规划历史遗留问题。提升城市品质。谋划实施城市功能区功能完善、城市集中供热、旧城区和城中村（棚户区）改造“三大行动计划”，大力推进城市综合提升、便民服务、交通畅通、城建环保、生态水系、生态绿化“六大重点工程”。沙河湿地公园建设通过国家验收。沙澧河二期、幸福渠市区段和“五路三桥”西环项目顺利开工，全面拉开生态水系建设和大交通体系建设的序幕。投资6亿元实施基础设施提升工程，改造主次干道47条、背街小巷2238条，城区新开工棚改项目6个，整治老旧小区996个，新安装交通护栏90公里，新施划停车位1.3万个，新建改造供热管网58公里、热力交换站15座。深入开展经营秩序、交通秩序、七小门店、卫生清洁等专项整治，城区卫生条件明显改善，城市通行和经营秩序有效改观。数字化城市管理平台和智慧城市建设顺利推进。县城和小城镇承载力不断提升，全市城镇化率达到50.91%，同比提高1.68个百分点。建设美丽乡村。“美丽宜居示范村”创建有序推进，基础支撑项目建设力度加大，争取美丽乡村项目22个，新建、改造农村公路360公里，新建电力线路1741公里，新建、完善农田防护林17万亩方、林业生态园区14个，郾城区、召陵区被确定为美丽乡村建设试点县（区），我市农村人居环境工作居全省第5位。

（五）民计民生持续改善。切实强化民生保障。扎实做好惠民利民各项工作，努力让广大群众得到更多实惠。民生支出129.9亿元，（上接1版）占一般公共预算支出的74.2%，省定十项民生工程和市定十件民生实事较好完成。脱贫攻坚深入推进。全市上下众志成城、真情投入、倾力帮扶，围绕精准识别、精准帮扶、精准脱贫，派出驻村第一书记990人、工作队员3052人，投入财政扶贫资金5.5亿元、金融扶贫资金12.84亿元，建成各类产业就业扶贫基地400余个，16779名贫困群众实现稳定脱贫，贫困村退出57个，舞阳县整体脱贫退出工作顺利通过省级核查验收。环保攻坚战果显著。各级各部门克难攻坚，各工业企业、施工企业顾全大局，PM10、PM2.5浓度均值实现双降，空气优良天数达到210天，空气质量改善率在全国338个地市中居第7位。我市成为全省唯一一个六项空气质量主要因子同比下降的城市。全面建立四级河长制度，7条省控河流断面达标率95.2%。城市饮用水源地水质达标率100%，沙河水体是全省最好的过境河流之一，城市黑臭水体基本消除。社会事业全面进步。努力办好人民满意教育，新建改扩建幼儿园63所、中小学25所，新增中小学学位7000个，56所农村薄弱学校办学条件得到全面改善。普通高考再创佳绩。漯河职业技术学院、漯河医专获批国家优质高等职业院校建设立项单位，漯河食品学院升本进展顺利。医疗卫生事业健康发展，市中心医院建成豫中南第一家生殖服务机构，郾城中医院病房大楼投入使用，我市被确定为公立医院综合改革首批国家级示范城市。体育、文化、旅游等社会事业繁荣发展，青少年示范性综合实验基地一期、文化馆建成投用，承办省级以上高水平体育赛事16项，“幸福漯河”系列群众文化活动被评为省级公共文化服务体系示范项目。城市交通更加便捷，新投入200台纯电动公交车，新开通公交线路3条，完成公交场站3座，市区公交票价统一调整为1元。社会保障能力不断增强。城镇新增就业4.4万人，失业人员再就业1.5万人。机关事业单位养老保险制度改革全面完成。城乡居民最低生活保障标准、农村“五保”集中和分散供养标准、城乡低保补助标准、社会散居和机构供养孤儿补助标准全面提高，养老、医疗、失业保险覆盖面进一步扩大。开展了大病补充医疗保险，为城乡低保家庭、建档立卡贫困家庭等建立起第三道医疗保障线。全国养老服务业综合改革试点工作扎实推进。全国适度普惠型儿童福利制度试点全面展开，两县三区全部纳入全国“百县千村”儿童福利工程。强化住房公积金扩面归集工作，建成保障性住房1.26万套，公共租赁住房实现常态化分配。社会治理更加有效。深化平安漯河建设，在全省成功打响扫黑除恶战役第一枪。应急预案体系和应急管理体制进一步完善。依法做好信访稳定工作。安全生产、食品药品安全、社会治安综合治理工作扎实有效，公众安全感指数居全省第4位，社会大局保持稳定。

一年来，我们不断加强民主法制建设，自觉接受人大法律监督、政协民主监督和社会监督，认真办理市人大代表建议和政协提案，支持民主党派、工商联和无党派人士参政议政。全面推进依法行政，强化审计监督，政府自身建设和治理能力不断提升。精神文明建设成效显著，群众获得感进一步增强。统计、人防、气象、司法等工作进一步加强，广播电视、新闻出版、史志档案、民族宗教、外事侨务、检验检疫、邮政通信等取得新成效，妇女、未成年人、老年人和残疾人合法权益得到保障。驻漯解放军、武警官兵和民兵预备役积极支持地方建设，军民融合发展进一步深化。

各位代表！这些成绩的取得，是在市委领导下，全市人民认真贯彻以习近平同志为核心的党中央一系列重大决策部署，全面落实省委、省政府工作要求，团结奋斗、顽强拼搏的结果。在此，我代表市人民政府，向工作在各条战线上的工人、农民、知识分子、干部，向驻漯解放军、武警消防官兵、公安干警，向所有关心支持漯河发展的各界人士，表示崇高的敬意和衷心的感谢！

充分肯定成绩的同时，我们也清醒地认识到，经济社会发展中还面临不少困难和问题：工业增长后劲不足，重大项目储备不多；企业创新能力不足、效益不佳，财税增收乏力；精准脱贫任务艰巨，环保形势依然严峻，企业风险、金融风险和政府债务风险不容忽视；城市基础设施和配套功能不够完善，城乡融合发展步伐不快；民生欠账较多，就业、养老、教育、文化、卫生、住房等公共服务水平与人民群众日益增长的美好生活需要还有较大差距；政府自身建设还存在薄弱环节，一些政府工作人员能力素质不适应新时代要求，主动服务、主动担当不够，执行力还需要进一步提升。对此，我们要采取针对性措施，认真加以解决。

二、新的一年工作安排

今年是全面贯彻落实党的十九大精神的开局之年，是决胜全面建成小康社会、实施“十三五”规划承上启下的关键一年。当前，世界经济复苏回暖，我国经济稳中向好，我们正处于大有可为的历史机遇期。党的十九大从战略全局上对党和国家事业作出规划和部署，为我们做好各方面工作提供了行动指南。中央将继续实施积极的财政政策并突出聚力增效，继续实施稳健的货币政策并突出中性适度，结构性政策将发挥更大作用，改革开放将加大力度，有利于我们保持经济持续健康发展。特别是经过长期不懈努力，漯河的发展水平站上了新的台阶，广大干部群众加快发展、不甘人后的愿望十分迫切，一大批企业在市场历练中凤凰涅槃，为今后的发展奠定了坚实基础。同时，我们也要客观认识经济社会发展中面临的困难和困难背后的深层次问题，特别是在经济转型、民生改善和要素制约方面所面临的压力。对此，我们既要正视困难、有效应对，更要抢抓机遇、乘势而上，坚持遵循规律、锲而不舍，奋力开创高质量发展新境界。

2018年政府工作的总体要求是：全面贯彻党的十九大精神，以习近平新时代中国特色社会主义思想为指导，坚持稳中求进工作总基调，坚持新发展理念，坚持高质量发展的根本方向，以供给侧结构性改革为主线，统筹推进“五位一体”总体布局和协调推进“四个全面”战略布局，全面发挥优势打好“四张牌”，按照新时代漯河经济社会发展“四三二一”工作布局，着力稳增长、促改革、调结构、惠民生、防风险，推动经济社会持续健康发展，在决胜全面建成小康社会、开启新时代漯河全面建设社会主义现代化新征程中迈出坚实步伐。

综合考虑发展趋势和工作导向，市委、市政府提出，今年全市经济社会发展主要预期目标为：经济保持平稳较快发展，生产总值增长8%，全社会固定资产投资增长9%，社会消费品零售总额增长11%，进出口保持平稳增长。发展结构持续优化，供给侧结构性改革取得扎实进展，一二三产业增加值分别增长3.5%、8%、10%以上，第三产业增加值占生产总值的比重进一步提高；规模以上工业增加值增长8.5%，高新技术产业占比进一步提升；常住人口城镇化率提高1.6个百分点。质量效益不断提升，一般公共预算收入增长8%，税收占比高于全省水平；研究与试验发展经费占生产总值的比重进一步提高；节能减排、环境保护等指标完成省下达任务。人民生活稳步改善，城镇新增就业3.5万人，城镇调查失业率和城镇登记失业率控制在省定范围以内，居民人均可支配收入增长8.5%，居民消费价格涨幅3%左右。

实现上述目标，要重点做好以下八项工作：

（一）着力深化工业供给侧结构性改革，提高发展质量效益。把发展经济的着力点放在实体经济上，加快发展方式转变、产业结构升级，持续提高供给体系质量。加快工业转型提质。坚持质量第一、效益优先，促进传统优势产业转型发展。以绿色安全、知名品牌为引领，以发展高附加值的功能性食品、保健性食品为重点，推动食品企业加强技术创新和新产品研发、拓展“智能制造”应用、加快品牌建设和集群发展，培育壮大龙头企业，巩固和扩大食品产业在全省、全国的领先地位。支持双汇集团完善本地产品结构、壮大本土生产规模，加快旺旺二期、统一二期等项目建设。抓好化工产业延链补链，发展精细化工，壮大产业规模，打造国内有影响的盐化工、钛化工基地。加快推进金大地真空制盐、合成氨搬迁改造和联碱生产项目。推动银鸽新上包装纸、高档生活纸及其后加工项目。全力推进漯河烟厂易地技改。支持以贾湖酒业为重点的白酒企业转型发展。加快推进长沙远大住工、安徽鸿路钢构等项目，积极创建全省绿色装配式建筑示范市。以利通液压、亿博橡胶等为龙头，壮大液压软管产业集群。以永光科技、昌泰电力等为龙头，打造电力器材产业基地。加快诚和通信设备三期、英利电子科技等项目建设，支持曙光健士、威森生物医药等重点企业发展，培育电子信息和生物医药产业。积极推动数据资源整合，谋划发展大数据产业。促进产业集聚发展。以提升主导产业、完善发展平台、激活管理机制为重点，实施产业集群提质、创新平台建设提升、产城融合发展提速专项行动，进一步完善基础设施、健全区内功能、壮大产业规模、提升产业层次，争创全省先进产业集聚区，力争全市产业集聚区内规模以上工业企业主营业务收入增长9%以上，百亿级产业集群达到5个。培育壮大实体经济。持续实施“十百千”亿级企业及产业集群培育工程、“小升规”工程、“小升高”工程和企业上市四大工程。积极推动制造业升级改造，提升供给产品的品种丰富度、品质满意度和品牌认可度。实施“互联网+”工业行动计划，示范引导企业利用互联网发展个性化定制、柔性化生产。支持智能车间、智能工厂建设，推进传统生产线数字化、智能化改造，推广“机器换人”应用项目。落实支持非公有制经济发展的政策措施。完善产权保护制度，严厉打击制假售假行为。弘扬企业家精神，营造尊重、支持、保护企业家干事创业的社会氛围，促进各类市场主体竞相发展。持续深化企业服务。建立企业服务例会，及时协调解决企业手续完善、生产经营、要素保障等方面的实际问题。严格落实经营服务性收费公示和涉企行政事业性收费“零收费”制度。加强煤电油气运热和资金协调，积极争取电力直接交易规模，切实降低企业要素成本。构建营商环境评价体系，创建全省营商环境建设示范市。

（二）着力扩大有效需求，推动经济行稳致远。持续释放有效需求，防范化解风险隐患，促进经济持续健康发展。扩大有效投资。全年谋划实施项目400个，总投资2600亿元，年度完成投资900亿元以上，带动全市完成固定资产投资1300亿元以上。安排重点项目160个，总投资1333亿元，年度完成投资530亿元。加强重点项目管理，完善新开工项目联审联批制度，落实一窗办理、容缺受理等机制。组织好项目集中开工、重点项目观摩等活动，促进重点项目快开工、快建设、快投产。建立完善重点项目建设推进例会制度，加强项目协调和后续服务，持续开展重点项目建设环境整治，为项目建设创造良好条件。培育壮大第三产业。充分挖掘消费领域潜力，加快发展现代服务业，推动服务业扩量提质。着力发展现代物流业，重点抓好双汇物流数字化冷链网络平台、申通物流产业园、圆通速递豫南区域总部等项目建设，培育壮大特色物流产业集群。加快发展现代金融业，积极引进股份制商业银行、保险、证券、基金、信托等金融机构和股权投资类企业，支持双汇财务公司等地方法人金融机构发展。大力发展电子商务，着力建设一批线上线下融合互动的新型专业市场。积极发展文化旅游和健康养老业，抓好龙江生态城文化大观园、贾湖国家遗址博物馆等项目建设，推进乡村旅游示范基地创建，加快市养老服务综合产业园等项目建设。培育壮大新兴服务业，发展总部经济、楼宇经济和研发设计、检验检测、会展商务等业态。认真落实房地产业健康发展的各项政策，加快建立多主体供给、多渠道保障、租购并举的住房制度，培育和发展住房租赁市场。强化载体支撑，推动生产性服务业向商务中心区集中，促进特色商业区专业化、集群化发展，力争“两区”内新增规模以上服务业企业15家、新增从业人员5000人，临颍、舞阳争创“二星”特色商业区。打好防范化解重大风险攻坚战。防范化解金融风险，严厉打击非法集资，妥善处理企业担保链、资金链风险；深化债权银行委员会制度，共同支持有市场、有效益的暂时困难企业；依法处置不良贷款，推进“债转股”市场化运作；加强信用体系平台建设和数据收集、应用，大力推进联合激励、联合惩戒。防范化解政府债务风险，全面开展政府债务审计，摸清真实底数，找准问题症结；制定风险防控和化解方案，稳妥化解存量；坚决查处违规举债、担保行为，严格控制增量；规范PPP项目；积极争取一般债券和专项债券。防范化解企业风险，对企业风险进行全面排查，分类管控，因企制宜，依法有效化解；帮助企业完善不动产登记手续，扩大抵押融资；支持企业规范法人治理结构，继续对企业上市和“新三板”、四板挂牌进行补贴，拓宽直接融资渠道；引导企业强化风险意识，加强财务管控，优化债务结构，降低财务杠杆。防范食品安全风险，健全食品安全信息收集发布和应急机制，严格食品市场准入和管理制度，完善新业态、新模式、新产品的监管方式和监管手段，确保不发生重特大食品安全事故和区域性、行业性食品安全风险。

（三）着力深化改革开放创新，持续蓄积发展势能。坚持以改革增红利、以开放拓空间、以创新促发展，不断累积促进发展的后发优势。增强改革推动能力。坚持凡是中央和省委部署的改革都要及时跟进落实，凡是能够先行先试的改革都要积极向上争取，凡是漯河发展需要的改革都要坚决主动推进。深入推进“放管服”改革，再取消一批行政审批事项、简化一批办事程序、削减一批审批公章。深化商事制度改革，试点开展“证照分离”改革，全面实施“双随机、一公开”监管。深化财税体制改革，培育涵养税源，强化税收征管，盘活存量资金，优化支出结构，努力提高财政保障能力。深化投融资体制改革，推动两大投融资集团加快市场化步伐。稳妥推进事业单位分类改革。加快承担行政职能事业单位改革。积极推进国有非工业企业改革。完成盐业体制改革。加快农村商业银行组建。抓好全国养老服务业综合改革试点。抓住我市纳入全省土地利用综合改革试点的机遇，加大“三块地”改革和土地收储力度。加快“互联网+政务”服务平台与实体大厅融合、行政权力事项和便民服务事项在线办理全覆盖，实现平台以外无审批。加快公共资源交易平台整合。推进杜曲现代家居产业集聚区和沙澧产业集聚区高铁以西市场化运作试点工作。深化食博会市场化改革，不断提高食博会的办会质量和影响力。增强开放带动能力。统筹对内和对外开放、引进来与走出去、扩大总量与提高质量，发展更高层次的开放型经济。完善对外开放平台。积极对接“一带一路”，跟进全省“三区一群”建设，加快与郑州航空港战略合作，培植开放新优势。加快漯河海关特殊监管区升级和海关漯河办事机构申建工作。对接省电子口岸，推广应用河南国际贸易“单一窗口”。创新招商运作机制。紧盯食品产业、装备制造业、战略性新兴产业、现代服务业等重点领域，完善产业图谱，建立项目储备库和目标客商资源库。持续深化驻地招商、以商招商、委托招商，推行“资本+产业”招商模式，探索市场化专业化招商，加强与行业龙头企业、行业协会、民间商会、投资咨询公司的战略合作，实现集群式引进、园区式共建。支持协鑫、华冠养元等在漯投资企业进一步增资扩股，扩大规模，建设区域性生产基地。健全招商推进机制。建立招商项目签约前的评估机制，算好产业账、税收账和就业账。优化招商工作考核，科学确定合同履约率、税收贡献率等指标权重。完善招商激励机制。让招商有功人员经济上得实惠、政治上有荣誉、社会上有地位。力争新签约超10亿元项目20个以上。增强创新驱动能力。大力培育创新龙头企业、“科技小巨人”企业，新增国家级高新技术企业5家以上，高新技术产业增加值增长30%以上。完善吸引、留住、激活人才的政策措施，创新柔性招才引智机制，着力培育和引进领军人才、创新团队，新培育5家市级以上创新型科研团队。加快创新平台建设，鼓励产业集聚区围绕主导产业建设共性技术研发中心。扎实推进商标品牌战略，争取国家商标受理窗口落户我市。培育区域双创基地，建设一批创客空间、创新工场等新型孵化载体。支持漯河职业技术学院建设“漯河市休闲食品协同创新中心”，支持漯河食品学院建设食品产业公共研发平台，新建省级工程技术研究中心3家以上。积极争取国家和省级科技创新项目，谋划实施一批市级科技创新项目。健全政产学研协同创新机制，鼓励企业组建产业技术创新战略联盟。全面实行“科技券”制度，着力提高科技支出占一般公共预算支出、全社会研发经费投入占GDP的比重。

（四）着力加快新型城镇化建设，提升城市品质品位。以创建全国文明城市为统领，以百城建设提质工程、城市“双修”和城建重点项目为抓手，全面提高城市规划、建设、管理、经营水平，不断增强城市承载力和辐射带动能力。强化规划引领。编制城市“双修”总体规划，修编绿地系统专项规划、城市供热专项规划，编修中小学布局专项规划。启动编制慢行系统、停车场、医疗卫生等专项规划。全力支持市人大常委会加快《漯河市城乡规划条例》立法工作。健全规划分级审批机制，处理好程序与效率的关系。深化文明城市创建。扎实开展新一轮全国文明城市创建活动，紧扣新的创建标准，统筹抓好城市和农村，推动文明城市创建扩面提标。以城市精细化管理为核心，进一步完善城管体制、整合城管资源、下沉城管责任，强化网格化管理。积极稳妥推进智慧城市建设，完善信息化平台，运用大数据提升城市精细化、智能化管理水平。加大城市管理综合执法力度，深化交通秩序、经营秩序、广告牌匾、空中管线、建筑工地五项整治，实施墙体立面、背街小巷、老旧小区、公厕、市民素质五项提升，抓好文明餐桌、文明旅游、文明交通、文明上网、城乡清洁五项行动。优化路名路牌、公交站牌、交通标识设置。注重城乡共建，深化移风易俗，提升农村精神文明建设水平。加快中心城区建设。持续实施“三大行动计划”，强力推进“六大重点工程”。今年安排城建项目236个，总投资609亿元，计划当年完成投资113亿元。新建改造南环路东延、大学路等30条市区道路，实施东入市口人民路、文化路综合提升工程，启动建设西环控制性工程及龙江路上跨京广铁路和下穿京港澳高速、107国道沙河桥、北舞渡沙河桥等改造工程；完成漯河船闸工程，启动马湾船闸工程、舞阳军李枢纽工程。有效推进小李庄（二期）、三里桥（二期）等省定棚改项目与国开行合作。开工建设澧河饮用水源地取水口上移工程。加快实施沙澧河二期综合整治、幸福渠市区段、示范区段沙河大二期、西城区生态水系、龙江生态城水系、沙澧河一期提升改造、沙澧河景区郾城五老台“两湖”区域综合治理等生态水系项目。统筹抓好公园游园建设、道路绿化提升等生态绿化工程，实施传统街区和历史文化建筑保护修复工程。推进百城建设提质。落实“以水润城、以绿荫城、以文化城、以业兴城”要求，抓好规划设计、功能完善、市容管理、城市经营四个关键环节。编制临颍、舞阳市政公用设施和公共服务设施专项规划，抓好两县重点城建项目建设，增强承载能力，争取两个县城常住人口分别增加1万人。抓好重点镇、特色镇建设，对10个市区周边镇实施镇容镇貌综合整治，建成宜居宜业新镇区，实现与中心城区融合发展；着力建设生态型和服务型小城镇，加快培育一批特色小镇。支持撤乡建镇，实现更高层次发展。树立城市经营理念，创新经营机制，探索自我增值、自我积累、自我投入、自我发展新路子。促进产城融合发展。以产业集聚区为突破点，实现产业集聚区总体发展规划、土地利用总体规划、城市总体规划、生态环境保护规划、区域公共服务设施规划无缝对接、精准套合。强化产业支撑，加快集聚区生产性服务业和生活性服务业发展。建立完善产城互动发展机制和共享机制，加快区内公共服务基础设施和配套功能建设，并与中心城区公共服务资源对接共享。提升产业集聚区生态环境，使其更加宜居宜创宜业。

（五）着力实施乡村振兴战略，推进农业农村现代化。按照“产业兴旺、生态宜居、乡风文明、治理有效、生活富裕”的总要求，科学编制实施乡村振兴战略规划，加大政策倾斜，强化扶持措施，让农业成为有奔头的产业，让农民成为有吸引力的职业，让农村成为安居乐业的美丽家园。深入推进脱贫攻坚。坚持精准扶贫、精准脱贫，确保扶持对象、项目安排、资金使用、措施到户、因村派人、脱贫成效“六个精准”。深入落实“三个五”政策措施，注重扶贫与扶志扶智结合、救急纾困与内生脱贫结合，改进帮扶方式方法，建立正向激励机制，下足“绣花”功夫，全面完成省定脱贫攻坚任务，巩固舞阳脱贫攻坚成效。抓好产业扶贫，深化金融扶贫，实施保险扶贫，让更多的贫困户在产业发展中得到实惠、在抵御风险中得到保障。持续深化农村改革。完善农村土地承包“三权分置”制度，在坚持落实集体土地所有权、稳定农户土地承包权前提下，平等保护土地经营权，切实保障农民的土地权益。深化农村集体产权制度改革，推进资源变资产、资产变股权、资金变股金、农民变股东等改革，激活乡村沉睡的资源。深化农村金融改革，支持金融网点、业务下乡。落实和完善融资贷款、配套设施建设补助、税费减免、用地等扶持政策，引导工商资本参与乡村振兴。支持乡村立足实际发展集体经济。支持各种专业人才通过各种方式服务乡村振兴事业。加快农业转型升级。坚持质量兴农、绿色兴农，深入推进农业供给侧结构性改革，加快由增产导向转向提质导向。按照藏粮于地、藏粮于技的要求，认真落实粮食安全市县长责任制，坚持保障粮食安全和提升农业经营效益并举，统筹推进千亿斤粮食田间工程、农业综合开发、土地整治、农田水利等项目，新建高标准粮田4万亩，粮食种植面积稳定在400万亩以上，粮食产量稳定在160万吨以上。持续调优种养结构，发展特色经济作物，扩大优质专用小麦、林果、蔬菜、苗木花卉种植面积。开展省级农产品质量安全县创建，实施农业标准化生产，大力支持农产品“三品一标”认证和区域公用品牌创建。深化双汇产业化工程，以现代畜牧业产业园区为载体，着力培育畜产品生产安全示范基地、高品质生猪供给基地、畜产品深加工基地。积极打造观光农业，开发休闲旅游、健康养生、生态教育等功能，创建一批生态旅游示范村镇和精品线路。深化农业科技提升，加强新技术、新品种引进推广，积极开展互联网+现代农业行动，建好用好益农信息社。创新农业经营体制，培育一批家庭工场、手工作坊、乡村车间。大力发展现代农业产业联盟，统筹培育新型农业经营主体，促进小农户和现代农业发展有机衔接。扩大农产品深加工比例，力争规模以上农产品加工营业收入突破1000亿元。提升乡村治理能力。强化农村基层组织建设，优化农村基层干部队伍结构，培养一支懂农业、爱农村、爱农民的“三农”工作队伍，提升指导服务“三农”的本领。健全自治、法治、德治相结合的乡村治理体系，探索乡村治理新模式，推进平安乡镇、平安村庄建设。着力改善农村人居环境。实施农村人居环境整治三年行动方案，以农村垃圾、污水治理和村容村貌、户容户貌提升为重点，建立完善垃圾收运体系和村庄保洁制度。扎实开展以“环境美、田园美、村庄美、庭院美”为主要内容的创建活动，力争建成一批村容整洁、环境优美、生态良好的美丽宜居乡村。大力推进农村“厕所革命”，加快实现无害化卫生厕所全覆盖。积极创建全国畜禽养殖废弃物资源化利用试点市，新培育一批生态美丽牧场，着力解决小散养殖场户“养殖扰民”问题。促进城乡深度融合。健全城乡融合发展机制，着力推动要素下乡、农民进城，促进资本、人才、科技、文化等进入乡村，为乡村振兴注入新动能。吸引已经在城市就业或生活的常住人口、城中村城郊村和产业集聚区内的人口、大中专院校毕业生、有意愿在城区落户的农民进城落户。深入推进城乡一体化示范区建设，积极探索城乡融合发展新样板。积极稳妥、分类处理新型农村社区遗留问题，推动乡镇所在地社区提标提质。

（六）着力强化污染防治，持续改善生态环境。坚守绿水青山就是金山银山的生态文明理念，以最严细的管控、最严格的监督、最严肃的追责、最严厉的处罚打好污染防治攻坚战，积极创造优良优美生态环境。深入推进大气污染防治。坚持依法治污、科学治污、精准治污、全民治污，确保完成省定目标，坚决打赢蓝天保卫战。全面实施月度生态补偿、绿色环保调度、污染物排放许可、排污权有偿使用和交易、企业环境信用评价等制度。落实“三治本三治标”要求，强化网格责任，突出重点时段、重点区域、重点行业，不断提升污染管控科学化精细化水平。大力开展城市清洁行动，全面实施建筑施工工地“七个百分之百”，有效抑制道路交通扬尘，严格落实各项禁烧禁燃措施。规划建设静脉产业园，推动各类垃圾分类处置、消纳利用。持续实施治本控源，加快调整能源消费结构，着力优化区域产业结构。强力推进水污染治理。全面落实河长制，坚持全域治理、源头治理，系统推进水污染防治、水生态保护和水资源管理。持续加大污水处理能力及管网配套建设，加快建设马沟污水处理厂、经开区污水处理厂二期、东城污水处理厂二期、舞阳第二生活污水处理厂、临颍第二污水处理厂扩建等工程，新增污水处理能力11万吨，努力实现城区生活污水全收集处理。基本完成舞阳、临颍两县黑臭水体截污纳管工作。城市饮用水源地澧河取水水质达标率保持100%，7条省控河流稳定达标。推进塔河、黑河、三里河水污染综合治理和生态修复，打造河流生态廊道，持续改善水环境质量。全面开展土壤污染防治。结合漯河实际，制定“1+4+8”土壤污染防治框架。全面开展农用地土壤污染状况详查，划定治理与修复区域；实施化肥、农药零增长行动，在试点区域开展农药包装废弃物、废弃农膜回收处理和生活垃圾分类处理；建立污染地块名录，实施农用地土壤环境分类管理和建设用地准入管理；制定土壤污染治理与修复规划，确保完成省定任务；完善土壤环境监测制度，加强土壤监测能力建设。提升绿色发展水平。推进资源全面节约和循环利用，加快循环经济产业发展。实施煤炭消费替代管理，严控新增煤炭消费，推动煤电减排升级，全部实现超低排放。推进全民节能行动，支持重点行业清洁生产和农村清洁能源建设。编制生态绿城建设规划和市区周边镇绿地系统规划，加快森林、湿地、流域、农田、城市五大生态系统建设。推进林业生态建设，积极争取国家储备林项目，在城镇周围和高铁、高速公路、干线道路、河渠两侧广泛布绿，沿重点河流建设水源涵养林，完善农田防护林体系。编制生态水系建设规划，加强湿地保护管理，完善提升沙河国家湿地公园，着力打造河畅水清地绿景美的中原水城、生态绿城。

（七）着力发展社会事业，提升公共服务水平。坚持抓重点、补短板、强弱项，着力优化基本公共服务供给。坚持教育优先发展。持续实施第三期学前教育行动计划。推动农村义务教育“全面改薄”，不断改善农村办学条件。扎实推进扩充城镇义务教育资源五年计划，新增中小学学位5000个，着力解决“择校热”“大班额”等突出问题，力争中小学超大班额比例下降到5%以内。支持漯河高中等优质教育资源建设分校，提升高中阶段教育质量。支持漯河工学院增加本科专业、扩大招生规模，加快推进漯河医专、技师学院新校区建设，支持食品学院升本，启动市一中专新校区建设，打造豫中南区域性职教中心。继续实施第二期特殊教育提升计划。支持和规范社会力量兴办教育。健全学生资助制度，努力让每个孩子享有公平而有质量的教育。深入推进全民健康。加快医疗卫生事业发展，提升基层医疗卫生服务能力，加强乡村医疗卫生机构标准化建设。深入推进分级诊疗、家庭医生签约服务和紧密型医疗联合体建设。加快区域性医疗中心建设步伐，重点抓好生殖遗传中心和心脑血管中心建设，建成市中心医院全科医师培训基地。巩固国家卫生城市创建成果，深入开展爱国卫生运动。提升计生服务管理水平。广泛开展全民健身，积极承接重大体育赛事，努力打造区域性体育赛事中心。加快发展文化事业。办好第四届许慎文化国际研讨会，提升“中国汉字文化名城”内涵。加强基层公共文化设施建设，扩大文化惠民工程，持续开展群众文化活动，加大“舞台艺术送农民”力度，促进公共文化服务均等化、常态化。继续实施文化产业三年行动计划，提升许慎文化、贾湖文化等特色文化品牌，推动重点文化项目建设。深入开展“扫黄打非”行动。礼拜五秘书网

（八）着力保障和改善民生，增进人民群众福祉。践行以人民为中心的发展思想，尽力而为，量力而行，办好一批民生实事，不断满足人民群众日益增长的美好生活需要。

办好一批群众急需、普遍受益的民生实事。一是持续改善贫困村和贫困人口的生产生活条件。完成省定农村贫困人口稳定脱贫任务；改造农村危房1048户；解决44个村7万人饮水安全问题；新建和改造农村道路100公里；对所有贫困村农村电网进行升级改造。二是继续提高城乡居民生活和妇女儿童健康保障水平。提高城乡低保对象月人均财政补助水平、特困人员基本生活标准、城乡居民基础养老金最低标准；继续免费开展出生缺陷产前筛查和新生儿疾病筛查；免费开展适龄妇女“两癌”筛查。三是大力发展便民服务。实现“沙澧一卡通”与全国200个城市互联互通，新建“环卫工人驿站”30个，开工建设双汇广场全民健身活动中心，完善100个村、社区、养老院室外健身设施。四是积极推进“厕所革命”。新建改建旅游公厕30座，在乡镇建成区、中心村新建改扩建公厕200座以上。五是继续推进城市集中供热。完成市区建成区30公里供热主管网铺设工作，在具备条件的居民小区推进集中供暖。六是大力发展学前、义务和职业教育。新建改扩建公办幼儿园47所，继续改扩建中小学项目10个，实施中小学“全面改薄”项目18个，新增职业教育校外实训基地30个以上。七是深化农村环境综合整治。确保60%的行政村达到农村人居环境达标村标准，40%的行政村达到农村人居环境示范村标准。八是持续加强城市基础设施建设。太白山路沙河桥和澧河桥、中山路上跨立交桥、牡丹江路沙河桥建成通车；打通泰山北路、解放北路等3条断头路；改造辽河路、嫩江路等人行道；改造提升泰山路、银江路等道路路灯；新建垃圾中转站5座；完成双龙、铁东和沙北部分区域雨污分流管网改造，扩容改建井冈山路等泵站；新建立体停车楼4座。九是着力扩大就业创业。城镇登记失业率控制在4.5%以内，动态消除“零就业家庭”，新增农村劳动力转移就业2万人，扶持农民工返乡创业5000人。十是扎实推进环境污染治理。全年大气优良天数达到220天以上，PM10浓度均值每立方米不高于100微克，PM2.5浓度均值每立方米不高于59微克，7条省控河流断面达到省定目标要求，城区黑臭水体治理全部达标。

持续提高就业质量。完善公共就业创业服务体系，深入实施职业教育攻坚和全民技能振兴工程，促进高校毕业生、城镇困难人员、退役军人等群体多渠道就业创业，推动农村劳动力转移就业，依法治理拖欠农民工工资问题。完成职业技能培训3.5万人次、农民培训1万人次以上。

加强社会保障体系建设。全面实行居住证制度，健全以居住证为载体的基本公共服务保障体系。落实好城乡低保、农村五保户各项补贴、残疾人“两项补贴”和孤儿补助政策，扎实做好特困人员供养和医疗救助、临时救助。全面实施全民参保计划，积极参与养老保险全国统筹。完善城乡居民基本医疗保险、大病保险、困难群众大病补充医疗保险和医疗救助制度，提高农村贫困人口门诊慢性病和门诊重特大疾病保障水平。推广政府购买居家养老社会化服务，提升医养结合试点工作质量。加强残疾人康复工作。健全农村留守儿童、妇女、老年人关爱服务体系。加快推进以农民工为主体的新市民群体的归集扩面工作，扩大住房公积金制度受益范围。

加强社会治理创新。扎实做好第九届村民委员会和第六届社区居民委员会换届选举工作。落实安全生产责任制，强化重点行业领域安全专项治理，坚决遏制重特大事故发生。深化“阳光漯河”行动，依法做好信访稳定工作。加强困难群体法律援助，推动刑事法律援助全覆盖。推进突发事件预警信息发布体系建设，提升防灾减灾救灾能力。加强社会治安防控体系建设，依法打击各类违法犯罪，坚决打好扫黑除恶人民战争，增强人民群众安全感，保持稳定和谐的社会秩序。

加强国防教育、国防动员和国防后备力量建设，支持驻漯解放军、武警部队、民兵预备役和人民防空建设，深入开展双拥共建，扎实推进军民融合，巩固军政军民团结。全面贯彻党的民族政策和宗教工作基本方针，促进民族团结、宗教和睦。支持工会、共青团、妇联等人民团体更好发挥桥梁纽带作用。加强经济运行监测和统计分析。做好外事侨务、史志档案、气象、地震、邮政、援疆等工作。

三、切实加强政府自身建设

面对新形势、新任务、新要求，我们必须把党的领导贯穿政府工作全过程各方面，着力打造忠诚、干净、担当的公务员队伍，全面增强政府执行力，努力建设人民满意的政府。

加强政治建设。把政治建设放在政府自身建设的首位，用习近平新时代中国特色社会主义思想武装头脑、指导实践、推动工作，切实增强“四个意识”，坚定“四个自信”，严守政治纪律和政治规矩，坚决维护党中央权威和集中统一领导，始终同以习近平同志为核心的党中央保持高度一致。认真开展“不忘初心、牢记使命”主题教育，深入推进“两学一做”学习教育常态化制度化。

坚持依法行政。深入推进依法治市，建设法治政府。坚持以宪施政、依法行政，严格依照法定权限和程序履行职责、规范行为。建立执法实践与普法教育相结合的工作机制，着力提高政府工作人员法治思维和依法行政能力。完善政府法律顾问工作机制，加强公职律师队伍建设，强化决策合法性审查，推进科学民主依法决策。落实行政执法责任制，强化服务型行政执法，严格规范公正文明执法。

主动接受监督。全面推进政务公开、信息公开，健全政府权力清单、负面清单、责任清单，强化对行政权力的制约监督。认真执行人大及其常委会的决议决定，落实重大事项向人大及其常委会报告、向政协通报制度。自觉接受人大法律监督、工作监督和政协民主监督、专门机关监督，切实强化行政监督和行政执法监督，虚心接受社会监督和媒体监督、舆论监督，广泛听取民主党派、工商联、无党派人士和人民团体的意见。提高办理人大代表建议、政协提案的满意率。

保持廉洁本色。扎实推进政府系统全面从严治党，切实履行党风廉政建设主体责任，全面落实“八项规定实施细则”和省委、市委各项要求，深入查找和纠正“四风”突出问题。加强党章党规党纪和法律法规教育，强化巡察和审计结果运用，持之以恒正风肃纪反腐，构建“亲”“清”新型政商关系，树立清正廉明的政府形象。

强化责任担当。提升担当的意识和责任，把敢于担当作为思想行动自觉和职业习惯；强化担当的能力和水平，既要政治过硬、还要本领高强，既要有专业精神、还要有专业能力，努力增强“八种本领”；创新担当的方法和艺术，善于研究法规、研究政策、研究机遇、研究问题，既讲规矩又讲创新，既讲程序又讲效率；营造担当的氛围和机制，有效治理为官不为和庸政懒政怠政，细化容错纠错免责机制，进一步提振政府工作人员的“精气神”。

提升执行能力。始终把执行力建设作为政府自身建设的重要内容，把雷厉风行和久久为功结合起来，以钉钉子精神做实做细做好各项工作。强化执行理念，勇于承担责任，主动进行沟通，拒绝各种推诿，抵制一切笼统，不找任何借口；严格执行标准，关注落实细节，确保执行不打折扣、不搞变通、不降标准；创新执行机制，实现决策部署要快、协同落实要快、结果反馈要快；完善执行评价，明确责任主体和责任边界，加强工作督查和责任调查，严格绩效考核与奖惩兑现，进一步营造攻坚克难抓落实、雷厉风行抓执行的干事创业氛围。

各位代表！使命呼唤担当，实干赢得未来。让我们更加紧密地团结在以习近平同志为核心的党中央周围，在省委、省政府和市委的坚强领导下，不忘初心、牢记使命，锐意进取、埋头苦干，为开启新时代漯河全面建设社会主义现代化新征程而努力奋斗！
